# Prospects for Protective Potential of *Moringa oleifera* against Kidney Diseases

**DOI:** 10.3390/plants10122818

**Published:** 2021-12-20

**Authors:** Tanzina Akter, Md Atikur Rahman, Akhi Moni, Md. Aminul Islam Apu, Atqiya Fariha, Md. Abdul Hannan, Md Jamal Uddin

**Affiliations:** 1 ABEx Bio-Research Center, East Azampur, Dhaka 1230, Bangladesh; tanzinatuli842@gmail.com (T.A.); atik.rahmanbt@gmail.com (M.A.R.); akhimoni840818@gmail.com (A.M.); aminul.btge@gmail.com (M.A.I.A.); atqiyafariha047@gmail.com (A.F.); hannanbmb@bau.edu.bd (M.A.H.); 2 Department of Biochemistry and Molecular Biology, Bangladesh Agricultural University, Mymensingh 2202, Bangladesh; 3 Graduate School of Pharmaceutical Sciences, College of Pharmacy, Ewha Womans University, Seoul 03760, Korea

**Keywords:** *Moringa oleifera*, antioxidant, anti-aging, fibrosis, inflammation, kidney disease

## Abstract

Kidney diseases are regarded as one of the major public health issues in the world. The objectives of this study were: (i) to investigate the causative factors involved in kidney disease and the therapeutic aspects of *Moringa oleifera*, as well as (ii) the effectiveness of *M. oleifera* in the anti-inflammation and antioxidant processes of the kidney while minimizing all potential side effects. In addition, we proposed a hypothesis to improve *M. oleifera* based drug development. This study was updated by searching the key words *M. oleifera* on kidney diseases and *M. oleifera* on oxidative stress, inflammation, and fibrosis in online research databases such as PubMed and Google Scholar. The following validation checking and scrutiny analysis of the recently published articles were used to explore this study. The recent existing research has found that *M. oleifera* has a plethora of health benefits. Individual medicinal properties of *M. oleifera* leaf extract, seed powder, stem extract, and the whole extract (ethanol/methanol) can up-increase the activity of antioxidant enzymes like superoxide dismutase (SOD), catalase (CAT), and glutathione (GSH), while decreasing the activity of inflammatory cytokines such as TNF-α, IL-1β, IL-6, and COX-2. In our study, we have investigated the properties of this plant against kidney diseases based on existing knowledge with an updated review of literature. Considering the effectiveness of *M. oleifera*, this study would be useful for further research into the pharmacological potential and therapeutic insights of *M. oleifera*, as well as prospects of *Moringa*-based effective medicine development for human benefits.

## 1. Introduction

Kidney diseases are considered among the major health problems worldwide. Acute kidney injury (AKI) is closely connected with chronic kidney diseases (CKD). Since 1990, CKD has been included in the list of non-communicable conditions investigated by the global burden of disease study. As the disease’s growth rate accelerates, it has become a global concern. The majority of incidents occur in low and lower-middle income countries [1,2,3]. The kidneys gradually lose their ability to function in CKD patients, and the glomerular filtration rate (GFR) falls below 60 mL/min per 1.73 m^2^ [1,2]. Mainly people who have been already suffering from diabetes, heart disease, or high blood pressure are at a high risk of developing CKD. Few drugs, such as prolyl hydroxylase domain inhibitors against anemia in CKD [3], can be used to treat CKD complications. The main pathologies involved in kidney complications are inflammation, oxidative stress, apoptosis, and fibrosis [4]. Unfortunately, no potential drug for treating kidney diseases exists at this time. Therefore, the search for a potential drug with fewer side effects to combat this disease is becoming increasingly important. *M. oleifera* Lam., also known as drumstick tree, is a *Moringaceae* family member that grows in the Indian subcontinent. This plant’s various parts have medicinal applications, such as antifungal, antiviral, anti-inflammatory, etc. [5,6,7,8]. *Moringa* leaves also have a low calorific value and can be included in the diet of obese individuals [9]. Furthermore, it contains numerous bioactive phytochemicals such as flavonoids, saponin, vanillin, omega fatty acids, carotenoids, ascorbates, tocopherols, beta-sitosterol, moringine, kaempferol, and quercetin that have been reported in its flowers, roots, fruits, and seeds, and can play a variety of roles in medicine [10,11,12,13]. In general, the choice of the most suitable bioactive substance for therapeutic purposes necessarily depends on the chemical formula of that specific compound, its structure giving its unique properties, and implicitly its mode of action [14]. Kaempferol has been shown to promote cancer cell apoptosis, such as MCF-7 and A549 cells [15]. Due to its anti-inflammatory and antioxidant properties, quercetin has the potential to be hepatoprotective, hypocholesterolemic, hypolipidemic, and anti-atherosclerotic [16]. *Moringa* has an anti-hyperglycemic effect, according to researchers who studied it in vivo on mice models [17].

Previous studies indicate that the juice of the super food *M. oleifera* enhances antimicrobial defense [18] and regulates insulin level, as well as glucose uptake in muscles [19,20]. Interestingly, *M. oleifera* showed a significant reduction of hyperglycemia, low-density lipoprotein (LDL) cholesterol, total cholesterol, fatty substances, FPG, and VLDL-cholesterol [21]. *M. oleifera* is also beneficial for skin, hair, liver, eye, blood pressure, treating anemia, kidney disease, and diabetes [22]. Several recent studies have documented the beneficial impacts of *M. oleifera* in alleviating renal diseases in animal model. Nafiu et al. [23] marked that gentamicin-induced impairment and oxidative stress significantly reduced by ethanolic extract of *Moringa oleifera* seeds in plasma, urine and kidney homogenate of rats. Akinrinde et al. [24] observed that *M. oleifera* extract attenuates the deleterious effects of renal ischemia-reperfusion through alleviation of oxidative stress. Soliman et al. [25] explored the ameliorative effects of *M. oleifera* against oxidative stress and methotrexate-induced hepato–renal dysfunction. Recently, Abu-Zeid et al. [26] discovered that the ecofriendly selenium nanoparticle using *M. oleifera* and/or *M. oleifera* ethanolic leaf extract reduces melamine-induced nephrotoxicity by alleviating of renal function impairments, oxidative stress, and apoptosis in rat kidney. Despite the great progress of *M. oleifera* in this field in recent years, less attention has been given to the effectiveness of *M. oleifera*, particularly against kidney related diseases. Therefore, there are still some issues which need further exploration, such as the protective effects of *M. oleifera* in kidney related disease difficulties and its prospects in drug development for human benefits.

This review updates the existing knowledge concerning the causative factors involved in kidney disease, as well as the therapeutic aspects of *M. oleifera*. Furthermore, this study provides a hypothesis on how *M. oleifera* would be effective in the anti-inflammation and antioxidant processes of the kidney, with the least amount of side effects.

## 2. Methods

**Figure 1:**
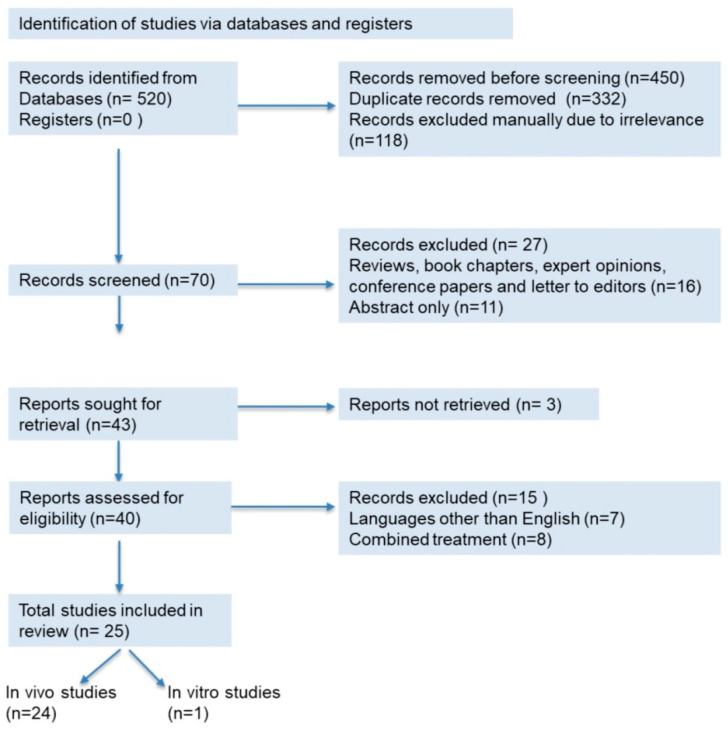
PRISMA 2020 flow diagram for the systematic review.

This systematic review was carried out following the Preferred Reporting Items for Systematic Reviews and Meta-Analyses (PRISMA) guidelines [27]. Databases such as Scopus, PubMed, and Google Scholar were accessed to retrieve information using the keywords ‘MeSH terms’, on ‘kidney diseases’ and ‘oxidative stress’ and ‘inflammation’, and ‘fibrosis’ and ‘*Moringa oleifera*’. The information was retrieved from 2011 to 15 June 2021. Automatic search tools were used to exclude some of the articles, while others were screened manually. Articles published in languages other than English were excluded. Reviews, book chapters, expert opinions, conference papers, and letters to editors were also excluded from this review. A total of 151 research articles were retrieved from the databases and discussed in this study (Figure 1). All information compiled in the table was obtained from these research articles.

## 3. Phytochemical Content and Pharmacological Potential of *M. oleifera* on Kidney Diseases

*M. oleifera* contains several bioactive phytochemicals including flavonoids and isothiocyanates [10]; polyphenols, carotenoids, alkaloids, and terpenoids [11]; and triterpenoids, moringyne, monopalmitic, di-oleic triglyceride, campesterol, stigmasterol, β-sitosterol, avenasterol, and vitamin A [12]. These bioactive phytochemicals are found in *M. oleifera* roots, fruits, and seeds. These phytochemicals have medicinal properties which have been shown to be effective antioxidant, antimicrobial, inflammatory, and anti-carcinogenic agents [28]. More studies are required to explore the role of bioactive phytochemicals specially in kidney diseases.

**Figure 2:**
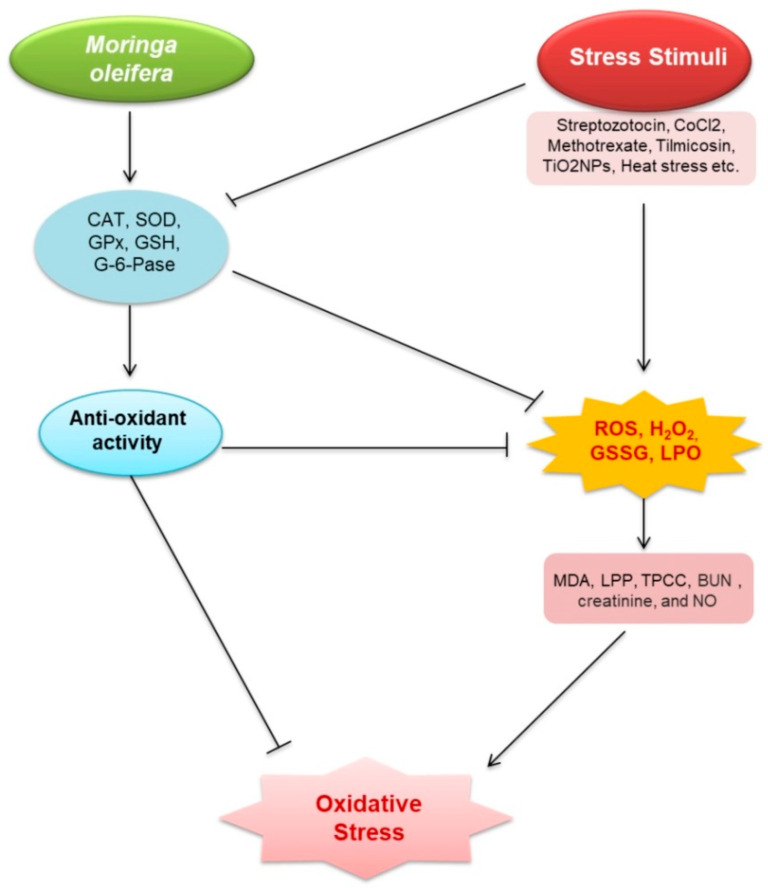
Renoprotective effects of *M. oleifera* against oxidative stress. Stress stimuli (streptozotocin, CoCl_2_, methotrexate, tilmicosin, TiO_2_NPs, acetaminophen (APAP), glycerol, and *Salmonella*) increased malondialdehyde (MDA), lipid peroxidation products (LPP), total protein carbonyl content (TPCC), blood urea nitrogen (BUN), creatinine, and nitric oxide (NO) production via triggering reactive oxygen species (ROS), H_2_O_2_, glutathione disulfide (GSSG), and lactoperoxidase (LPO). Oxidative stress emerged as a result of these events. MO—induced models, on the other hand, increased the expression of catalase (CAT); superoxide dismutase (SOD); glutathione peroxidase (GPx); glutathione (GSH), total antioxidant capacity (TAC); delta-amino levulinic acid dehydratase (ALAD), and G-6-Pase, which then activates glutathione (GSH). These stressors inhibit the expression of oxidative stress suppressive factors. ROS, H_2_O_2_, GSSG, and LPO, all related to oxidative stress, were decreased by GSH. GSH is also capable of reducing oxidative stress.

**Figure 3:**
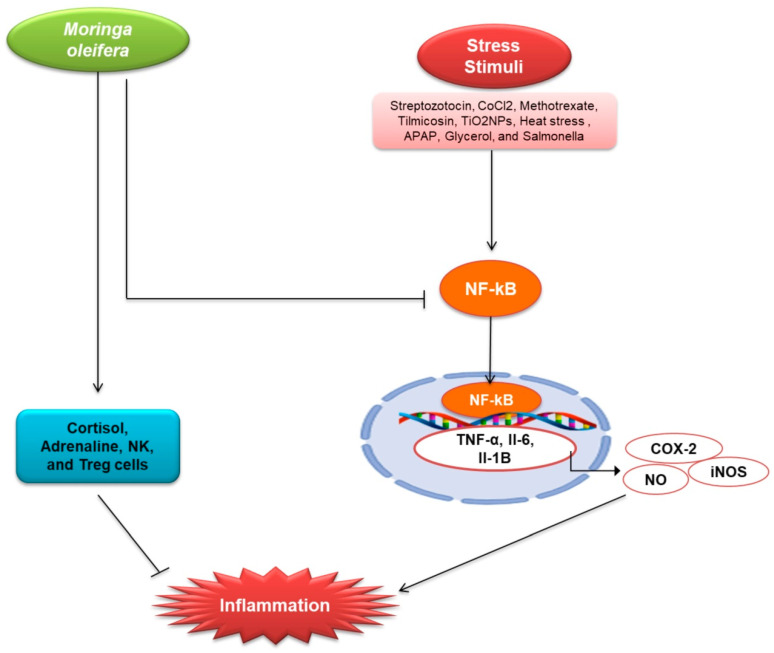
Renoprotective effects of *M. oleifera* against inflammation. The expression of C-reactive protein (CRP), which activates NF-kB in the cytosol, is linked to stress factors. TNF-, Il-6, Il-1B, iNOS, and COX-2 are all activated when NF-kB enters the nucleus and binds to DNA. All of these elements have been linked to the development of inflammation. NO is activated even more by iNOS. NO is thought to be a pro-inflammatory mediator that causes inflammation. In the cytosol, *M. oleifera* suppressed the expression of CRP and NF-kB. It also boosted cortisol, adrenaline, NK, and Treg cells, which helped reduce inflammation. Anti-inflammatory hormones Cortisol and Adrenaline Both NK cells and Treg cells are anti-inflammatory regulators.

*M. oleifera* also possesses a variety of pharmacological properties, which are closely associated with the presence of its bioactive compounds. Therefore, in the following section we highlighted the pharmacological potential of *M. oleifera*. *M. oleifera* showed pharmacological potential against some plausible factors such as oxidative stress, inflammation, fibrosis, and other pathologies responsible for kidney diseases. The potential effects of *M. oleifera* against risk factors associated with kidney disease in the following sections as shown in Figure 2 and Figure 3.

### 3.1. Oxidative Stress

Oxidative stress is caused by an imbalance between the excessive free radical generation and insufficient antioxidant defense [29,30]. It is frequently observed in CKD [31,32,33], and has become a diagnostic factor [34]. A number of studies documented that *M. oleifera* has antioxidative properties to protect and/or alleviate cellular damage (Table 1 and Figure 2). *M. oleifera* extracts and compounds, particularly quercetin, kaempferol, isothiocyanates, rutin, myricetin, ascorbic acid, and β-carotene, showed antioxidant potentials either via direct scavenging of free radicals [35].

Methanol extract of *M. oleifera* reduced the oxidative stress in STZ induced male rats by lowering the production of MDA, ROS, LDL, and CHOL, which increase the risk of CKD [36,54]. Methanol extract also lowered the generation of MDA, AOPP, NO, H_2_O_2_, GPx, and GST, all of which induce oxidative stress in ischemia-induced Wistar rats [29]. Another study showed that metabolic extract reduced the levels of BUN and creatinine, and total protein is increased in CKD patients [42]. Ethanolic extract of *M. oleifera* inhibits oxidative stress and atherosclerosis in CKD by lowering LDL [20]. 8-OHdG causes oxidative stress to DNA and promotes cancer [56], ameliorated by the ethanolic extract of *M. oleifera* [56]. Ethanol extracts decrease the plasma creatinine level by enhancing the process of creatinine clearance [30]. Plasma sodium and potassium levels were raised after treating nickel-induced Wistar rats with ethanolic extract of *M. oleifera* [34]. Ethanolic extract detoxified plasma by reducing the bilirubin levels (indirect/direct), urea levels, etc., in ML-induced male Sprague Dawley rats [48]. HO-1 and Nrf2 expression were stimulated by leaf extract of *M. oleifera* at dosages of 300 and 400 mg/kg body weight, respectively [25,41]. Leaf extracts up-regulated the level of total thiol TiO_2_NPs induced male albino rats, which play an important role in antioxidant protection [41]. Leaf extract of *M. oleifera* also downregulated the oxidative stress generating mediators in sodium fluoride (NaF)-induced *Oreochromis niloticus*, gentamicin-induced rabbit, and APAP-treated mice [23,42,57].

*M. oleifera* alcoholic extract reduced oxidative stress by lowering the lipid peroxidation, and ROS in iodide injected rabbits [51]. Furthermore, fermented leaf extract of *M. oleifera* boosts the antioxidant activity in bacteria-induced mice [53]. *M. oleifera* extract reduced the manifestation of MDA, indicating that the free radicle overproduction was reduced in both Tilmicosin and Hg induced rats. Abarikwu et al. showed that SOD level was increased after treatment with *M. oleifera* in tilmicosin induced rats [40]. Hydroalcoholic root extract raised blood sugar, antioxidant enzyme activities, and G-6-phase activities, which protect the kidney from nephropathy in Beryllium-induced rats [45]. Seed powder reduced free radical species, TPCC, metal content, and increased ALAD activity in lead-treated rats [57]. In arsenic-treated rats, seed powder of *M. oleifera* considerably increased antioxidant function including GSH, CAT, and ALAD [46].

### 3.2. Inflammation

The kidney is responsible for maintaining whole-body homeostasis. Kidney disease is characterized by inflammation as a major pathology [58,59,60]. Acute or chronic disease such as ischemia, toxins, or inflammation affects kidney tubules, causing kidney fibrosis that is associated with reduction of GFR in kidneys [61]. Kidney injury is linked to the production of cytokines levels, which prolongs the acute phase of kidney disease [62]. Moreover, chronic inflammation is regarded as a comorbid condition in CKD [63]. Many plants have an anti-inflammatory action through active substances such as hesperidin, diosmin, withaferin, fucoidan, thymoquinone, etc. [64,65,66,67]. Here, the anti-inflammatory effects of *M. oleifera* has been discussed. *M. oleifera* has been reported to exhibit strong inflammatory activity (Table 1 and Figure 3). Methanolic extract of *M. oleifera* reduced inflammation in STZ induced male Wister rats by down-regulating the tumor necrosis factor (TNF-α), IL-6, and MCP-1, an important chemokine [36,54]. Tang et al. investigated the effects of ethanolic extract of *M. oleifera* in metformin-induced mice and observed that the *M. oleifera* declines the production of inflammatory markers and the expression of cyclooxygenase-2 (COX-2) and nitric oxide synthase (iNOS) by reducing the phosphorylation of mitogen-activated protein kinase (MAPK) pathway [20]. Ethanolic extract of *M. oleifera* down-regulates the inflammatory cytokines in CoCl_2_-induced rats, including NO, which is involved in the pathogenesis of inflammation [37]. Leaf extract of *M. oleifera* inhibits inflammatory cytokines production and regulates the inflammation by inhibiting NF-kB [25]. It was also observed that inflammation in Tilmicosin (Til) induced rats was reduced by *M. oleifera* extracts [39]. *M. oleifera* leaf extract protects against interstitial kidney inflammation with fibrosis by down-regulating KIM-1 in TiO_2_NPs induced male albino rats [41]. *M. oleifera* extract increases the secretion of cortisol, adrenaline, Treg cells, NK, and leptin, promoting anti-inflammatory cytokines and regulating the immune system [47]. *M. oleifera* treatment reduced the expression of KIM-1, TIMP-1, and TNF-α in ML-induced male Sprague Dawley rats [48]. TNF-α, an inflammatory cytokine that stimulates IL-1; IL-6, downregulated by *M. oleifera* in Seabream (*Sparus aurata*); and activated TGF-β, elicits anti-inflammatory effects [49]. *M. oleifera* also reduced the inflammatory cytokines in APAP-treated mice, where APAP induces AKI [50]. Fermented extract of leaves also reduces the Nrf2 in *Salmonella*-induced mice [53].

Moringa seed’s phytochemicals can reduce the production of nitric oxide (NO) and the gene expression of LPS-inducible iNOS and interleukins 1β and 6 (IL-1β and IL-6) compared to curcumin [68]. Flavonoids have been shown to be effective inhibitors of nitric oxide synthase type 2 (NOS-2) actions, and it also inhibits protein tyrosine kinase action that is involved in the NOS-2 expression at the molecular level [69,70,71]. Flower extract can cause the activation of pro-inflammatory proteins such as toll-like receptors. In the flowers, quercetin and kaempferol can inhibit the signal transducer and activator of transcription 1 (STAT-1) and the NF-*κ*B pathways [72,73]. *M. oleifera* flowers contain 80% hydroethanolic, a potent agent of anti-inflammation in the NF-*κ*B signaling pathway [74]. Scientists discovered that phenolic glycosides suppress inducible iNOS expression and NO production in mouse macrophage cells, as well as COX-2 and iNOS proteins [75,76]. *Moringa* extracts eventually down-regulate the inflammatory mediators because its seeds and flowers contain many bioactive compounds. Each of these compounds has its individual effects.

### 3.3. Fibrosis

Kidney fibrosis is defined as a radical harmful connective tissue deposition on the kidney parenchyma, which leads to renal dysfunction. Epithelial to mesenchymal transition (EMT) is the main mechanism of kidney fibrosis, and the TGFβ-1-SMAD pathway and hypoxia are known as the main modulator of EMT [32,77]. TGF-β-induced expression of fibronectin, type I collagen, and PAI-1 rat kidney fibroblast cells is reduced by *M. oleifera* extract [55]. Furthermore, moringa root extract selectively inhibited TGF-β-induced phosphorylation of SMAD4 and ERK expression. These results suggest that moringa root extract may reduce renal fibrosis by a mechanism related to its antifibrotic activity in rat kidney fibroblast cells. Oral administration of *M. oleifera* seed extract reduced CCl4-induced liver fibrosis in rats [78].

### 3.4. Other Pathologies Those Are Associated with Kidney Diseases

Autophagy has a critical role in kidney physiology and homeostasis [79], and, thus, its regulation is an important determinant of kidney diseases [61]. AKI or CKD causes mitochondrial damage, but damaged mitochondria begin to accumulate in response to these types of stimuli. Autophagy protects the kidney through the removal of ROS-producing mitochondria [80,81,82]. Apoptosis is a type of programmed cell death in which cells are killed by a controlled system. It is an energy-dependent complex process [83]. It contributes to develop AKI, even organ failure [84]. Ischemia/reperfusion (I/R) induces apoptosis or necrosis in the kidney and loss of tubular cells, leading to decreased GFR [85,86]. Renal tubular cells express cell surface ‘death receptors’ of TNF-α which is responsible for inducing apoptosis [87]. Also, ROS production in kidney disease is responsible for promoting apoptosis [86].

TNF-α inducer of apoptosis, also increased the expression of apoptosis-related molecules which was down-regulated by ethanol extract of *M. oleifera* in CoCl_2-_treated rats [37,88]. Leaf extract at a dose of 300 mg/kg body weight reduced the expression of caspase-9, the precursor of caspase-3, leading to apoptosis [25,89]. Bcl-2 inhibited apoptosis by blocking cytochrome c release and preventing caspase activation [90] while it was up-regulated by ethanol extract of *M. oleifera* in ML-induced rats. *M. oleifera* also reduced the expression of TIMP-1, which is involved in renal fibrosis and apoptosis [48].

## 4. Prospects for *M. oleifera* in Drug Development

Researchers are targeting the development of drugs from natural sources instead of the synthetic drug because natural sources have fewer side effects than synthetic sources. Nigerian scientists proved that *M. oleifera* is a beneficial herb and causes no harm to the body and kidneys [91]. Another study reported that higher doses of *M. oleifera* created toxicity in rats, but a moderate level dose of *M. oleifera* is safe [92]. *M. oleifera* has been shown to alleviate diabetic nephropathy in alloxan-induced rats [93]. Acetaminophen causes hepato-renal toxicity, which can be cured by *M. oleifera* treatment at the dosage of 500 mg/kg [94]. *M. oleifera* reduced necrosis, dilatation of renal tubules in Cd-induced rats, where Saleh et al. suggested that *M. oleifera* could be used as an herbal drug [95]. *M. oleifera* leaf extracts reduced oxidative stress, kidney, and liver damage [96]. A randomized placebo-controlled study suggested that *M. oleifera* leaf capsules can be used to control blood sugar level and blood pressure level [97]. Moreover, aqueous extracts of *M. oleifera* can reduce metal (As (III), Cd, Ni and Pb) toxicity and showed the protective effects in *Saccharomyces cerevisiae* [98].

The rich phytochemical profile and advances in biotechnological techniques have made this tree indispensable for opening a new era in medical science. An in vitro propagation technique provides new insights into developing more effective, eco-friendly, and biodegradable products using mass multiplication and production techniques. Though efficiency in in vitro propagation techniques for *M. oleifera* has been established, there are still gaps in the production of metabolites and those specific metabolites in the human body. The use of biotechnological approaches will help in the commercialization of important plant products. There is no doubt that biotechnological protocols will allow great research to make *M. oleifera* one of the essential solutions for various health issues including kidney diseases.

## 5. Conclusions

**Figure 4:**
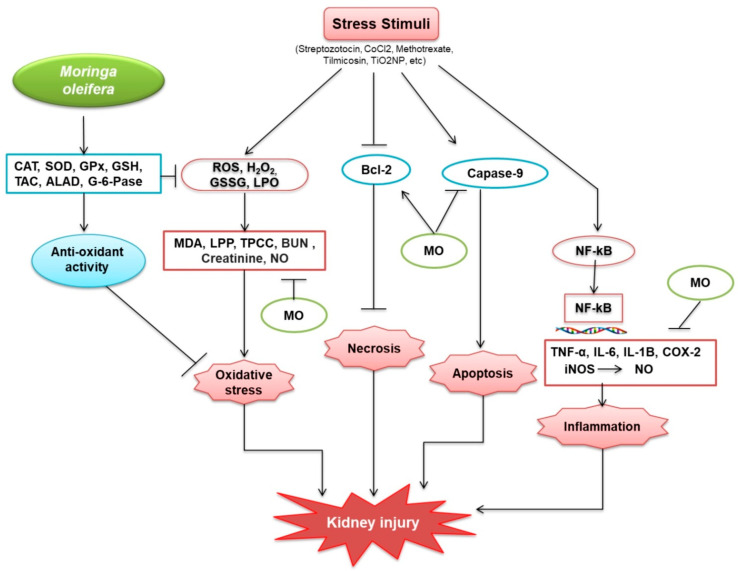
Protective mechanisms of *M. oleifera* against kidney injury. *M. oleifera* increased the production of catalase (CAT); superoxide dismutase (SOD); glutathione peroxidase (GPx); glutathione (GSH); total antioxidant capacity (TAC); delta-amino levulinic acid dehydratase (ALAD); and G-6-Pase, which facilitated oxidative stress reduction by activating glutathione (GSH), a non-protein thiol that suppresses free radicals. GSH suppresses the oxidative stress situation. *M. oleifera* also suppressed oxidative stressors caused by ROS, H_2_O_2_, GSSG, and LPO by inhibiting MDA, LPP, TPCC, BUN, Creatinine, and NO. Bcl-2 was similarly produced by stress stimuli and was linked to the suppression of necrosis, induced by *M. oleifera*. *M. oleifera* inhibited the expression of Caspase-9, a protein involved in the formation of caspases. Following NF-kB, stress stimuli also increased CRP expression. NF-kB then moved from the cytosol to the nucleus, bound to DNA, and activated inflammation-related proteins. *M. oleifera* inhibited the mechanism by which inflammation factors were produced, hence, reducing inflammation. *M. oleifera* has been linked to a reduction in the progression of kidney disease.

Kidney function declines with age, and aging-related kidney complications proportionately increase. Their side effects limit the effectiveness of existing drugs for treating kidney diseases and, therefore, natural compounds with fewer side effects are being evaluated. The literature discussed in this review suggests that *M. oleifera* alleviates several pathological factors associated with kidney diseases, including inflammation and oxidative stress. However, a mechanism associated with protective potential of *M. oleifera* against kidney diseases has been provided in this study (Figure 4).

This study discusses the insights of *M. oleifera* against kidney diseases including AKI and CKD, which have not been reported previously. In addition, further studies are needed to confirm the effects of the bioactive phytochemicals (vitamins, alkaloids, polyphenols, isothiocyonates, glucosinolates, tannins, and saponins) of *M. oleifera* against kidney diseases. We anticipate that the points raised in this review will provide a future research direction for understanding how pharmacological interventions based on natural products could modulate kidney disease. In contrast, it would shed light on how *M. oleifera*-based drugs would potentially be a kidney protective agent in treating aging-associated kidney abnormalities. Considering the harmful effects of synthetic resources and their non-renewable nature, the use of natural resources as a source of medicine has received a lot of attention in recent years. *M. oleifera* based medicine would be an excellent protective agent against several risk factors associated with kidney diseases.

## Figures and Tables

**Table 1:** Summary on the protective effects of *M. oleifera* against kidney diseases.

Sl. No.	Experimental Model	Treatment Dose of Moringa Extract	Major Research Outcomes	Molecular Markers	Ref.
1	STZ-induced nephrotoxic male Wister rats	250 mg/kg b wt for 6 weeks	Amelioration of oxidative stress and inflammation	↓MDA and ROS ↑CAT, SOD, GSH, and GPx ↓TNF-α and IL-6	[36]
2	*db/db* mice	150 mg/kg/day for 5 weeks	Oxidative stress and inflammation	↓LDL ↓TNF-a, ↓IL-1b, ↓IL-6, ↓COX-2, and ↓iNOS	[20]
3	Ischemia-reperfusion induced Wistar rats	200 mg/kg for 7 days; 400 mg/kg, 7 days by flank incision	Oxidative stress	↓MDA, ↑PC, ↓AOPP, ↓NO, ↓H_2_O_2_, ↓GPx and GST, ↑GSH	[24]
4	CoCl_2_-induced rats	Orally received 400 mg/kg bw/day for 6 weeks	Oxidative stress Inflammation and Apoptosis	↓MDA, ↓H_2_O_2_, ↓8-OHdG, ↓CRP, ↓MPO, ↓TNF-α, and ↓NO ↓TNF-α, and NO↓	[37]
5	Gentamicin (GENT) induced Wistar rats	Orally treated with 100, 200 and 400 mg/kg/day for 28 days	Oxidative stress	↓K^+^ level, ↓plasma creatinine, ↑Creatinine clearance, ↓MDA, ↑SOD	[23]
6	Nickel-induced Wistar rats	5% *M. oleifera* 10% *M. oleifera* 15% *M. oleifera*	Oxidative stress	↓plasma creatinine, ↓urea, and ↑potassium, ↑plasma level of sodium	[38]
7	Methotrexate (MTX)-induced Mice	300 mg/kg body weight, orally for 7 days	Oxidative stress Inflammation Apoptosis	↓urea and ↓creatinine, ↓total protein, ↓MDA, ↑SOD and ↑GSH, ↑HO-1, ↑Nrf-2 ↓NF-kB, ↓Caspase-9	[25]
8	Tilmicosin (Til) induced Sprague Dawley rats	400 or 800 mg/kg bw, by oral gavage for 7 days	Oxidative stress, inflammation	↓H_2_O_2,_ ↓MDA, ↑SOD, ↑GPx, mRNA expression ↓TNF-α, ↓IL-1β	[39]
9	Hg-induced Male Wistar rats	1.798 mg/kg p.o three times per week for 21 days	Oxidative stress	↓MDA level, ↑SOD, and ↑CAT	[40]
10	TiO_2_NPs induce male albino rats	Daily oral dose of 400 mg/kg b w for 60 days	Oxidative stress Inflammation	↓MDA, ↑SOD, ↑GSH, ↑GST,↑GPx, ↑Total thiol and ↑HO-1, ↑Nrf2 ↓KIM-1, ↓NF-кB, ↓TNF-α, and ↓HSP-70	[41]
11	NaF induced *Oreochromis niloticus*	6.1 mg/L for 8 weeks	Oxidative stress	↓MDA, ↑SOD, ↑CAT, ↑GSH, ↑GPx, ↑TAC	[42]
12	Gentamicin-induced (80 mg/kg) Rabbit	150 mg/kg body for 10 days, 300 mg/kg wt. for 10 days	Oxidative stress	↓Serum urea and creatinine levels, ↓LPO	[43]
13	Lead treated Male Wistar rats	500 mg/kg for 7 days	Oxidative stress	↓ROS, ↓LPP, ↓TPCC, ↓metal content,	[44]
15	Beryllium-induced rats	150 mg/kg daily for 5 weeks	Oxidative stress	↓LPO, ↑GSH, ↑antioxidant enzymes activities, ↑G-6-Pase activity	[45]
16	Arsenic-induced toxicity in rats	500 mg/kg, orally, once daily	Oxidative stress	↑ALAD, ↑GSH,↓ROS, ↑SOD, ↑Catalase, ↓GSSG	[46]
17	Heat stress (HS)-induced rabbits	100, 200, and 300 mg, 6 weeks	Inflammation	↑cortisol, ↑adrenaline, ↑leptin, ↓IFN-γ, ↓TNF-α, ↓urea, and ↓creatinine, ↓IL-10, ↑NK, and ↑Treg	[47]
18	ML-induced male Sprague Dawley rats	Orally 800 mg/kg bw 800 mg/kg bw	Oxidative stress, Inflammation Apoptosis	↓Total bilirubin, ↓direct bilirubin, ↓indirect bilirubin, ↓urea, and ↓creatinine ↑serum levels of protein, ↑albumin, ↑globulin, ↑GPx, and ↑CAT ↓KIM-1, and ↓TNF-α and ↑Bcl-2, ↓TIMP-1	[48]
20	Seabream (*Sparus aurata*)	10% *M. oleifera* 4 weeks	Inflammation	↓TGF-β and ↓TNF-α ↑ACH_50_ and ↑lysozyme activities and ↑IgM level ↑ (lyso and c3), ↑ (occludin and zo-1)	[49]
21	APAP-treated mice	100 mg/kg of bw, 200 mg/kg bw	Oxidative stress, inflammation	↑SOD, ↑CAT and ↑GPx, ↓MDA, ↓TNF-α, ↓IL-1β, ↓IL-6, ↓IL-10	[50]
22	Iodide injected Rabbit	50 mg/kg body weight, orally once daily for 27 sequential days	Oxidative stress	↓MDA, ↑GSH, ↓NO, ↓lipid peroxidation, ↓ROS	[51]
23	Glycerol induced rat	50 mg/kg and 100 mg/kg for 7 days	Oxidative stress Inflammation	↑SOD, ↑GST, ↑GP_X_, ↑GSH ↓MPO, ↓Creatinine, ↓BUN, ↓NO ↓H_2_O_2_, ↓AOPP, ↓MDA, ↓PC,↑PT, ↑NPT,↓KIM-1 and ↓NF-ҝB	[52]
24	Salmonella-induced mice	14, 42 and 84 mg/kg/day for 28 days	Oxidative stress inflammation	↑HO-1, ↑SOD-2 ↑Nrf-2	[53]
25	STZ-induced rats	250 mg/kg and SRC. 42 days	Oxidative stress inflammation	↓LDL, ↑HDL, ↓CHOL, ↑ORAC ↓IL-6, ↓TNF-α, and ↓MCP-1	[54]
26	TGF-β-treated rat kidney fibroblast cells	10, 50, and 100 µg/mL	Fibrosis	↓Type I collagen, fibronectin, and PAI-1 ↓TβRII and Smad4, and phospho-ERK	[55]
27	Gentamicin-induced Wistar rats	28 days at graded doses of 100, 200 and 400 mg/kg	Nephrotoxicity	↓Creatinine and MDA ↑SOD	[23]

MDA, Malondialdehyde; TNF-α, tumor necrosis factor-alpha; IL-6, interleukin-6; STZ, streptozotocin (C8H15N3O7); GSH: glutathione; CAT, catalase; SOD, superoxide dismutase; GPx, Glutathione peroxidase; IL-1β, Interleukin 1 beta; COX-2, cyclooxygenase-2; iNOS, Inducible nitric oxide synthase; AOPP, advanced oxidation protein products; PC, protein carbonyls; NO, nitricoxide; H_2_O_2_, hydrogen peroxide; 8-OHdG, 8-hydroxy-2-deoxyguanosine; MPO, myeloperoxidase; CRP, C-reactive protein; MTX, methotrexate; HO-1, heme oxygenase-1; Nrf2, nuclear factor erythroid 2-related factor 2; TAC, total antioxidant capacity; LPP, lipid perioxidation products; TPCC, total protein carbonyl content; ALAD, delta-amino levulinic acid dehydratase; BUN, Blood urea nitrogen; KIM-1, transmembrane tubular protein; Bcl-2, B-cell lymphoma 2; TGF-β, transforming growth factor beta; CHOL, Cholesterol; ORAC, oxygen radical absorbance capacity; and APAP, acetaminophen. ↑, increased; ↓, decreased.

## Data Availability

Not applicable.
